# Chitosan can stop or postpone the death of the suckling mice challenged with foot-and-mouth disease virus

**DOI:** 10.1186/1743-422X-7-125

**Published:** 2010-06-12

**Authors:** Dong Li

**Affiliations:** 1 Key laboratory of Animal Virology of Ministry of Agriculture, National Foot-and-Mouth Disease Reference Laboratory of China, State Key Laboratory of Veterinary Etiologic Biology, Lanzhou Veterinary Research Institute, Chinese Academy of Agricultural Sciences, Lanzhou, Gansu, 730046, China

## Abstract

In the study, a method called "hardening in liquid phase" for preparing chitosan granules with glutaraldehyde as crosslinker and Tween 80 as surfactant and paraffin liquid as dispersant was established. The chitosan granules were light yellow and insoluble in water or oil, but they swelled in acid solution and narrowed in neutral or alkaline solution. Furthermore, some of characteristics of the chitosan granules were revealed. **(a) **Stability: Their shapes were stable at pH 7.0 and pH **8.0 **and -30°C~120°C. The shelf life is at least one year in vitro at room temperature. **(b) **Safety: Some experiments of their lethal effect to suckling mice and pathogenicity to mature mice proved the chitosan granules were harmless. **(c) **Antiviral activity: Some suckling mice injected with chitosan granules were still alive or delayed death compared with control group when they challenged with foot-and-mouth disease virus (FMDV). Such anti-FMDV capacity could maintain 1 week and was the strongest on the third day.

## Findings

Chitosan (deacetylated chitin) is derived from chitin, the component of the cell walls of fungi, the shells of insects, and especially crustaceans. Thus, it is can be serviced relatively inexpensively from widely available materials, which is the second most abundant polysaccharide in nature. Chitosan is the commonly used name for poly-[1-4]-β-D-glucosamine [3].

As we know, the available antibody will be induced in 1 week after the antigens or vaccines enter animal's bodies. The pathogens can invade bodies easily in the empty period without specific immune defense. Some immunopotentiators can remedy such limitation. Chitosan is a good example. In this report, a method called "hardening in liquid phase" to prepare chitosan granules were established and some of characteristics such as the stability and the safety and the anti-FMDV activity of the chitosan granules were revealed.

Chitosan was bought from Zhejiang yuhuan chemical company, China. The virus strains were O/CHN/99(LD_50 _7.0) and Asia 1/JS/05(LD_50 _6.0).

Five gram of chintosan were dissolved in 200 ml of 2%(v:v) acetic acid with 10 ml tween-80, stirred continuously for 2 hrs, added 2 ml of 25% (v:v)glutaraldehyde, stirred rapidly for 5 min. and poured this Mixture into 500 ml liquid paraffin, stirred with High-speed magnetic stirrer for 1 hr to prepare chitosan granules. Then poured them into sand core funnel for vacuum pumping, washing them with petroleum ether, Isopropanol, Methanol, deionized water in turn, and stored at 2% glycine solution.

The chitosan granules were put into six different solutions pH 3, pH4, pH 5, pH 6, pH 7, and pH 8 to observe their change of form, 10 ml in each same streptomycin bottle at room temperature. One week later, the chitosan granules in pH 3, pH4, and pH 5 were swelling and suspending and the height (13 mm) were almost same; the chitosan granules in pH 6 were swelling and suspending slightly but the height (3 mm) were lower; the chitosan granules in pH 7 and pH8 were deposited in the bottom of the bottle, and the height were 2 mm. when the solution pH8 were modulated to pH4 for one day, the granules swelled. Instead, when the solution pH4 were modulated to pH8 for one day, the granules shrank, which means they are reversible. Furthermore, the chitosan granules in solutions pH = 7 or =8 were stable for at least one year at room temperature, but unstable in solutions pH < 6, which became loose in 3 months. When these six chitosan granules were alternate freezing and thawing at -30°C overnight for three times, the granules in solutions pH 7 and pH 8 were stable but in solutions pH <6 were unstable. When these six chitosan granules were treated at 120°C for one hour for 3 times, similarly, the granules in solutions pH 7 and pH 8 were stable but in solutions pH < 6 became loose.

In order to test the safety of chitosan granules, they were injected to 25 suckling mice of 4-day-old, 0.1 ml (contain 0.5 mg) each. One week later, they were healthy. Similarly, the granules were injected to 10 mature mice of 8-week-old, 2 ml (contain 10 mg) each. One month later, they were normal and healthy.

Three groups of suckling mice of 4-day-old were used to test the anti-FMDV activity of the chitosan granules. In the first group, ten suckling mice were injected 0.1 ml of the chitosan granules (contain 0.5 mg) and 0.1 ml of O/CHN/99 strain at the same time, and other ten suckling mice were only injected 0.1 ml of O/CHN/99 strain as the control. The death times of ten suckling mice were (hours):20.0, 21.0, 21.5, 22.0, 23.0, 24.0, 24.0, 24.0, 25.0, 25.5. The average death time was 23.0 hours. The death times of ten controls were (hours):19.5, 19.5, 20.0, 21.0, 21.5, 21.5, 21.5, 21.5, 21.5, and 21.5. The average death time was 20.9 hours. Thus, the average death time of the experimental suckling mice were delayed 2.1 hours compared with control animals. After *t *test, 0.01 < P < 0.025, the difference was significant.

In the second group, ten suckling mice were injected 0.1 ml of the chitosan granules but 0.1 ml of O/CHN/99 strain were challenged after three days, and other ten suckling mice were only injected 0.1 ml of O/CHN/99 strain as the control. The death times of ten suckling mice were (hours): 22.0, 22.5, 23.0, 23.5, 23.5, 24.0, 24.5, 24.5, 25.5, and 26.0. The average death time was 23.9 hour. The death times of ten controls were (hours):19.5, 19.5, 20.5, 20.5, 21.0, 21.0, 21.5, 21.5, 21.5, and 21.5. The average death time was 20.8 hours. So the average death time of the experimental suckling mice were postponed 3.1 hours compared with controls. After *t *test, P < 0.001, the difference was very significant.

**Figure 1:**
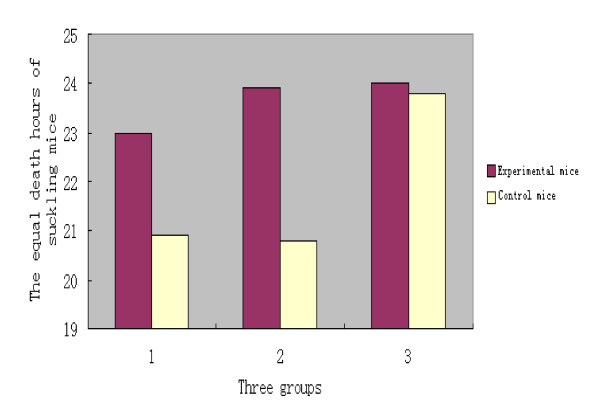
**The comparisons of average death hours of suckling mice challenged with FMDV O/CHN/99 strain after injected with chitosan granules at the zero day, the third day and the seventh day**.

In the third group, ten suckling mice were injected 0.1 ml of the chitosan granules but 0.1 ml of O/CHN/99 strain were challenged after seven days, and other ten suckling mice were only injected 0.1 ml of O/CHN/99 strain as the control. The death times of ten suckling mice were (hours): 21.5, 21.5, 23.0, 24.0, 24.0, 24.5, 25.0, 25.0, 25.5, and 26.0. The average death time was 24.0 hours. The death times of the controls were (hours):21.0, 22.0, 22.5, 23.5, 24.0, 24.5, 24.5, 25.0, 25.5, and 25.5. The equal death time was 23.8 hours. Therefore, the average death time of the experimental suckling mice were postponed 0.2 hours compared with controls. After *t *test, P > 0.5, the difference was not significant. The results of three groups were showed in table 1 and figure 1.

**Table 1:** The death situation of suckling mice challenged with FMDV O/CHN/99 strain at the zero day, the third day, and the seventh day after injected with chitosan granules

		The death hours of the suckling mice challenged with FMDV O/CHN/99 strain										Average(hr)	Survived numbers
Group 1	Experimental mice	20.0	21.0	21.5	22.0	23.0	24.0	24.0	24.0	25.0	25.5	23.0	0
	Control mice	19.5	19.5	20.0	21.0	21.5	21.5	21.5	21.5	21.5	21.5	20.9	0
Group 2	Experimental mice	22.0	22.5	23.0	23.5	23.5	24.0	24.5	24.5	25.5	26.0	23.9	0
	Control mice	19.5	19.5	20.5	20.5	21	21	21.5	21.5	21.5	21.5	20.8	0
Group 3	Experimental mice	21.0	22.0	22.5	23.5	24.0	24.5	24.5	25.0	25.5	25.5	24.0	0
	
	Control mice	21.0	22.0	22.5	23.5	24.0	24.5	24.5	25.0	25.5	25.5	23.8	0

We changed the challenged FMDV strain from O/CHN/99 to Asia 1/JS/05, and still set three groups. There were five suckling mice in each group, and other five were as control (see table 2). Five suckling mice in each group were injected 0.1 ml of the chitosen granules, and they were challenged with 0.1 ml of Asia 1/JS/05 strain at the first day, the third day and the seven day respectively. The average death time of the experimental suckling mice were postponed 1.2 hours in the first group. Two of the suckling mice were survived in the second group. The average death time of the suckling mice were postponed 0.4 hours in the third group. Therefore, the death situations of suckling mice were similar while challenged them using two different serotype FMDV strains. It showed that the chitosan have anti-FMDV activity, which could maintain 1 week and was the strongest on the third day.

**Table 2:** The death situation of suckling mice challenged with Asia 1/JS/05 at the zero day, the third day, and the seventh day after injected with chitosan granules

		The death hours of the suckling mice challenged with FMDV Asia 1/JS/05 strain					Average(hr)	Survived numbers
Group 1	Experimental mice	21.5	22.0	23.0	23.0	24.0	22.7	0
	Control mice	19.5	21.0	22	22.5	22.5	21.5	0
Group 2	Experimental mice	24.0	26.0	29.0	-	-		2
	Control mice	20.5	21	22	23	23	21.9	0
Group 3	Experimental mice	22.5	22.5	23	23.5	25	23.3	0
	Control mice	22	22	23	23	24.5	22.9	0

China circulated FMD type O in 1999 and type Asia 1 in 2005. The serious economic loss was made. Vaccination is the main prevent and control policy in China and other developing countries. Generally, the IgM isotype of antibody is the first detectable serum neutralizing antibody appearing at 3 to 4 days following FMDV infection or vaccination. Maximum serum IgM responses are observed around 10 to 14 days post-infection, after which the response declines. Serum IgGs can be detected as early as 4 to 7 days post-infection or vaccination. These are the isotypes which become the major neutralizing antibodies by two weeks after encounter with the virus antigen [2]. Thus, there are at least 0-4 days empty times without neutralizing antibody. The innate immune defenses are activated immediately after infection, more rapidly than the specific responses, and the host is more reliant on innate defense during the first days after vaccination. The elevated innate defenses could delay the onset of disease symptoms, giving the specific immune defenses the necessary time to develop a more durable protection. Immunostimulants can change the immune status of animals to improve their ability of defense disease. Chitosan was studied as an immunostimulants 20 years ago due to its harmless, biodegradable, biocompatible, abundant and anti- antimicrobial. And now, chitosan is widely used in human and veterinary medicine [4]. The data showed that chitosan strongly modulates the functional activity of the auxiliary cells involved in immune responses, such as macrophages and granulocytes. In this report, we produced chintosan granules because phagocytized particles of chitin and chitosan (but not their soluble analogues) increase the generation of active oxygen species in mouse (*Mus musculus *L.) alveolar macrophages and induce the synthesis of γ-interferon in cultured C57BL/6 mouse splenocytes caused by interaction of primed macrophages with natural killer cell [5]. Thus, the ability of chitin and chitosan to induce interferon synthesis can be an additional important factor of antiviral resistance. In mice, chitosan strongly increased the local and systemic immune responses (production of IgA and IgG antibodies) to influenza A (Texas H1N1) and B (Panama) viruses coadministered with an antigen (purified hemagglutinin and neuraminidase)[1]. Thus, chitosan can affect the induction phase of immune responses in animals and many effecter mechanisms of the immune system.

Two different serotype FMDV strains were used to observe the activity of chitosan. Two suckling mice were survived when challenged with Asia 1/JS/05 at the third day, but the death time were delayed without alive mice when challenged with O/CHN/99. The possible reason was that the virus titer of Asia 1/JS/05 was lower than which of O/CHN/99. Anyway, it was confirmed that the chitosan has the activity of anti-FMDV in our experiment.

## Competing interests

The author declares that they have no competing interests.

## Authors' contributions

DL has made substantial contributions to conception and design, acquisition of data, analysis and interpretation of data; has been involved in drafting the manuscript or revising it critically for important intellectual content independently.
